# Pubertal Pathways in Girls Enrolled in a Contemporary British Cohort

**DOI:** 10.1155/2010/329261

**Published:** 2010-06-22

**Authors:** Krista Yorita Christensen, Mildred Maisonet, Carol Rubin, Adrianne Holmes, W. Dana Flanders, Jon Heron, Jean Golding, Michael A. McGeehin, Michele Marcus

**Affiliations:** ^1^Epidemiology Department, Rollins School of Public Health, Emory University, 1518 Clifton Road NE, Atlanta, GA 30322, USA; ^2^Environmental Epidemiology and Population Health Research Group, Research Centre of the University of Montreal Hospital Centre (CRCHUM), 3875 rue Saint-Urbain 3e étage, Bureau 314 Montréal, QC, Canada H2W 1V1; ^3^National Center for Environmental Health, Centers for Disease Control and Prevention, 4770 Buford Hwy, Atlanta, GA 30341, USA; ^4^Department of Social Medicine, University of Bristol, 39 Whatley Road, Bristol BS8 2PS, UK; ^5^Department of Community Based Medicine, Centre for Child and Adolescent Health, University of Bristol, Barley House, Oakfield Grove, Clifton, Bristol BS8 2BN, UK

## Abstract

Data from the Avon Longitudinal Study of Parents and Children were used to describe initiation of secondary sexual characteristic development of girls. Tanner stages of breast and pubic hair and menarche status were self-reported via mailed questionnaires, administered from ages 8–14. Initiation pathway was categorized as breast [thelarche] or pubic hair [pubarche] development alone, or synchronous. Average ages at beginning breast and pubic hair development were estimated using survival analysis. Factors associated with initiation pathway were assessed using logistic regression. Among the 3938 participants, the median ages at beginning breast and pubic hair development were 10.19 (95% CI: 10.14–10.24) and 10.95 (95% CI: 10.90–11.00) years. Synchronous initiation was the most commonly reported pathway (46.3%), followed by thelarche (42.1%). Girls in the pubarche pathway were less likely to be obese or overweight at age 8 or have an overweight or obese mother. Girls in the thelarche pathway were less likely to be of nonwhite race or be the third born or later child.

## 1. Introduction

In females, the first visible signs of pubertal development are breast and pubic hair development [1, 2]. These processes are governed by two separate physiologic systems (the hypothalamic pituitary gonadal axis and hypothalamic pituitary adrenal axis, resp.), so breast and pubic hair development does not necessarily occur at the same time, or at the same rate of progression. A majority of girls are thought to experience relatively synchronous pubarche and thelarche, with the first appearance of pubic hair and breast budding occurring within a few months of each other (or observed at the same clinic visit). However, some girls will begin pubarche without corresponding thelarche and vice versa [3–6]; this is often referred to as asynchronous development. Different initiation pathways may reflect differential exposures, both external environmental exposures and exposure to endogenous hormones, and timing of pubertal milestones may impact future health outcomes including risk for overweight/obesity and breast cancer [5, 7–9]. Despite the importance of the initiation of puberty, the process and factors which impact pathway and timing have not been well described in a contemporary cohort.

Among participants in the Fels longitudinal study in the United States (1948), the majority of girls (85.8%) experienced asynchronous development, with over half of girls entering breast development before pubarche [10]. A Swedish study (1976) found that while 47% of girls experienced synchronous breast and pubic hair development, nearly half (45%) of girls had thelarche preceding pubarche [3]. An opposite pattern was found in a Swiss study (1983), where over half of girls (53%) were reported to have experienced pubarche without breast development [4]. A more recent (2003) study in the US using clinical assessments of pubertal stage found that 51.6% of eligible girls experienced asynchronous development [5]. Further, those girls who experienced thelarche as the initial visible marker of pubertal development had an earlier age at menarche compared to girls who experienced pubarche as the initial marker (12.6 years and 13.1 years, resp.) and were also more likely to have higher BMIs before and throughout puberty. Some reasons for the differences between studies with regards to pathway distribution may include a biased sampling frame, sample size, and method for pubertal staging. However, these studies suggest that asynchronous initiation of secondary sexual characteristic development may be relatively common and an important predictor of age at menarche and future health outcomes. Puberty, as well as menarche, is occurring earlier than in the past, based on data from the late 1800s to present [11–14]. Timing of maturation is important, as accelerated development has psychological and physiological consequences, including earlier age at first sexual activity, increased risk-taking behavior (including smoking, drinking, and use of illicit drugs), increased depression, and decreased physical activity [15–19]. Early age at menarche is also associated with increased risk of adult obesity [7, 8] and of breast cancer [20]. In this study, data from a contemporary, longitudinal cohort were used to describe initiation of secondary sexual characteristic development.

## 2. Materials and Methods

The Avon Longitudinal Study of Parents and Children (ALSPAC) is a prospective cohort study of ~14,000 pregnant women residing in Avon (United Kingdom [UK]) who had an expected delivery date between April 1, 1991 and December 31, 1992 [21]. Details of the study design have been published and are available online [21, 22]. A “Growing and Changing” questionnaire was developed to collect information on pubertal development; the questionnaire was distributed to participants at the ages of 8, 9, 10, 11, 13, and 14 years (1999–2005) [21, 23]. Questionnaires were not distributed at age 12. For all ages, parents or guardians could choose to complete the questionnaire themselves, complete it with their child, or allow the child to complete it herself. Menarche status (and if appropriate, age at menarche) was determined via self-report. Physical development was assessed using diagrams of Tanner stages [6]; these diagrams were developed at the University of North Carolina's Population Center and have been previously validated and successfully used in other cohort studies [24–26]. Each characteristic (breast and pubic hair development) has 5 possible stages, ranging from prepubertal (stage 1) to fully developed (stage 5). The respondent then selected the stage which most closely aligned with the girl's current stage of development. 

For these analyses, the cohort consisted of female singletons completing at least one puberty questionnaire in the appropriate age range, as determined using age at questionnaire completion (i.e., only girls 8 years of age included for the 8 year old questionnaire). Girls who reported a certain stage of breast or pubic hair development on one questionnaire then reported a lower stage of development on a subsequent questionnaire were excluded from these analyses. Girls who reported achieving menarche on a questionnaire, but reported not yet having their first period on a subsequent questionnaire, were also excluded. If more than one age at menarche was given, the first reported age at menarche was used for analysis on the assumption that it was the most accurate report. If reported age at menarche was greater than age at questionnaire completion, age at menarche was treated as missing.

Initiation pathway was determined by identifying the first instance in which a girl reported being in stage 2 or higher for breast and/or for pubic hair development. If the initial report of being in a stage ≥2 was reported for only breast development or for only pubic hair development, the girl was categorized as being in the thelarche or the pubarche pathway, respectively. If the initial report of being in stage ≥2 was for both breast and pubic hair development, the girl was categorized as being in the synchronous pathway. Consequently, the girls with synchronous development (i.e., reported moving beyond stage 1 for breast and pubic hair in the same questionnaire) entered stage 2 in the time elapsed since the last questionnaire, which in most cases was a year. 

Median ages at entry into breast stage 2 and pubic hair stage 2 were determined using parametric survival analysis, assuming a normal distribution. Various other distributions were used for comparison, and similar results were found; therefore, only the normal distribution results are reported here. Since the ALSPAC data were collected on an annual basis, the exact age at which a girl entered a stage of breast or pubic hair development is not known. Rather, it is known what stage a girl is in at a particular point in time (i.e., at the time each questionnaire is completed), leading to interval censoring. If the start of the interval is not known, data are considered left censored, and if the end of the interval is not known (i.e., the event is not observed), data are considered right censored. The LIFEREG procedure in SAS accommodates right, left, and interval censoring and was used to perform all parametric survival analyses. Age at menarche was compared between initiation pathways using a regression model with initiation pathway included as a predictor. In order to avoid potential downward bias when estimating age at entry into breast and pubic hair stage 2 by pathway, girls with unknown initiation pathway (*n* = 417) were randomly allocated to the 3 initiation pathway groups (proportionate to the distribution among girls with known pathway) and included in the survival models for age at entry into breast and pubic hair stage 2. 

Polytomous logistic regression models were used to identify maternal and child characteristics associated with initiation pathway (thelarche, pubarche, or synchronous). The candidate variables were identified from the literature as being associated with pubertal development (i.e., breast and/or pubic hair development, and/or menarche), and included mother's prepregnancy body mass index (BMI), mother's age at delivery, mother's age at menarche, mother's educational level, mother's social class, child's birthweight, child's race/ethnicity, child's birth order, and child's height and BMI at 8 years of age. Social class was derived using the 1991 Office of Population Censuses and Surveys [27]. Upper class consisted of classes I (professional occupations) or II (managerial and technical occupations); middle class of classes IIINM (nonmanual skilled occupations) or IIIM (manual skilled occupations); and lower class of classes IV (partly skilled occupations) or V (unskilled occupations). Height and BMI at 8 years of age were obtained from clinic visit data when available (71.3% and 66.9% of girls, resp.), and from self-report at the 8-year Growing and Changing questionnaire where clinic data were not available (3.4% and 5.6% of girls, resp.). Percentiles of height and BMI for month of age were defined using female-specific growth standards from a representative sample from the UK [28]. The association of each candidate variable with initiation pathway was assessed using a polytomous logistic regression model, using a cutoff of *P* < .30. Then, each variable that met this criterion in the univariate analysis was assessed for effect modification, by testing all 2-way interaction terms with each other covariate separately in polytomous logistic regression models. All variables that were associated with pathway in the univariate analysis and any relevant interaction terms were then entered into a multivariate logistic regression model. A *P*-value of  .05 was used to identify characteristics that remained associated with pathway in this model, using a stepwise selection method. Finally, the analysis was repeated excluding BMI as a covariate, due to the large number of girls missing information on BMI at 8 years of age (27.5%). We did not adjust for multiple comparisons.

Human subject protection was assessed and approved by the ALSPAC Law and Ethics Committee, the Local Research Ethics Committees, and the CDC Institutional Review Board.

## 3. Results

**Figure 1:**
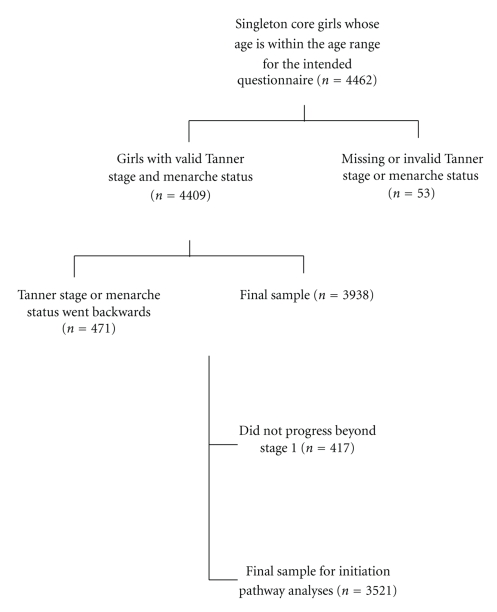
Flowchart of eligibility and exclusions.

In the ALSPAC cohort, 4462 singleton girls completed and returned at least one Growing and Changing questionnaire in the correct age range. Of these, 4434 had submitted information on breast development, and 4427 on pubic hair development, for at least one questionnaire. A small number of girls returned inconsistent reports of menarche status (*n* = 17). More girls had inconsistent reports of breast development (*n* = 320, 7.2%) or pubic hair development (*n* = 223, 5.0%), for a final sample size of 3938 girls (Figure 1). Initiation pathway could not be determined for some individuals, who did not progress beyond Tanner stage 1 for breast and pubic hair development (*n* = 417); these participants were excluded from pathway-specific analyses.

 Nearly all (96%) of girls were white, and the majority of girls were born to a mother with an O-level education (roughly equivalent to a United States high school diploma; 35.2%) or higher (40.1%). Just over two thirds of the girls were the first (34.6%) or second (33.3%) born. The mean maternal age at delivery was 28.6 (SD = 4.6) and ranged from 15 to 44. Most of the girls were in the normal height and BMI ranges for their age in months at age 8 (78.2% and 74.7%, resp.). Approximately one quarter were either overweight (12.4%) or obese (10.4%) at age 8. 

Most girls reported beginning breast and pubic hair development in the same questionnaire (synchronous pathway; 46.3%), or beginning breast development in advance of pubic hair development (thelarche pathway; 42.1%). The remaining girls (11.6%) reported pubic hair development preceding breast development (pubarche pathway). The median age at entry into stage 2 of breast development was 10.19 (95% CI: 10.14, 10.24) years, while the median age at entry into stage 2 of pubic hair development was 10.95 (95% CI: 10.90, 11.00) years. Girls in the synchronous pathway had a median age at entry into stage 2 of 10.65 (95% CI: 10.58, 10.72) years for breast and for pubic hair development (Table 1). The median ages at initiation for the thelarche and pubarche pathways were slightly younger (9.43 [95% CI: 9.35, 9.51] years and 9.44 [95% CI: 9.28, 9.59] years, resp.). 

In this cohort, 62.7% of girls had achieved menarche by the time of the 14-year questionnaire; age at menarche was missing for 208 (8.4%) of these girls. The median age at menarche estimated from the parametric survival analysis model was 12.87 (95% CI: 12.82, 12.91) years, and reported age at menarche ranged from 7.6 years to 14.9 years (Table 1). Using a life-table approach, the median age at menarche was 12.92 (inter-quartile range: 12.08, 13.67), and the mean was 12.86 (SE = 0.02). Age at menarche differed by initiation pathway. For girls in the synchronous pathway, median age at menarche was 12.84 (95% CI: 12.78, 12.91) years; girls in the thelarche pathway had a similar median of 12.78 (95% CI: 12.71, 12.85) years, while girls in the pubarche pathway had a slightly later median age of 13.13 (95% CI: 13.00, 13.26) years.

Factors associated with initiation pathway in univariate logistic regression analysis were girl's BMI at 8 years of age, girl's ethnic background, girl's birth order, mother's educational attainment, mother's age at delivery, and mother's prepregnancy BMI (Table 2). In the multivariate polytomous logistic regression model, variables which remained associated with initiation pathway were girl's BMI at 8 years of age, girl's ethnic background, mother's prepregnancy BMI, and birth order; no interaction terms remained in the final model (Table 3). Girls in the thelarche pathway were most likely to be overweight or obese, followed by girls in the synchronous pathway. Girls in the thelarche pathway were also less likely to be of nonwhite race compared to girls in the synchronous or pubarche pathways. Similarly to results for girl's own BMI, girls in the pubarche pathway were the least likely to have overweight mothers. Finally, girls in the thelarche pathway were less likely to be the third born or later child, compared to girls in the synchronous pathway. 

In the model excluding girl's BMI as a covariate, variables which remained associated with initiation pathway included mother's prepregnancy BMI, mother's age at delivery, girl's ethnic background, and birth order. Results were similar to those for the full model, with the addition that older maternal age at delivery, 25–29 years of age or ≥30 of age, was more common for girls in the pubarche pathway compared to girls in either the synchronous (OR = 1.72 [95% CI: 1.14, 2.60] and OR = 1.62 [95% CI: 1.06, 2.46], resp.) or thelarche pathway (OR = 1.97 [95% CI: 1.31, 2.98] and OR = 1.71 [95% CI: 1.12, 2.60], resp.).

## 4. Discussion

Initiation of secondary sexual characteristic development was assessed for girls participating in the ALSPAC study. To our knowledge, this is the first study to assess pubertal pathways in a longitudinal cohort using self-reported data. Nearly half (46.3%) of girls reported beginning breast and pubic hair development at the same time; this is similar to a recent US study [5], and to an earlier Swedish study [3]. However, among girls who reported asynchronous entry into breast and pubic hair development, the proportion of girls entering puberty through the thelarche pathway in the US cohort was slightly lower compared to the present study (65.7% versus 78.4%). Compared to an earlier Swiss study [4] and findings from the Fels longitudinal study in the US [10], our findings are somewhat different, with a smaller proportion of girls experiencing synchronous initiation. This may be due to differences in sociodemographic and physiological characteristics (including body composition), as well as the use of a different rating system. In the Swiss cohort, the authors stated that their method of visual inspection may have missed early signs of breast development, although the reasons for this were not given [4]. 

 We estimated median ages at entry into breast and pubic hair stage 2 to be 10.19 and 10.95 years. Girls in the pubarche and thelarche pathways entered puberty somewhat earlier (9.4 years), compared to girls in the synchronous pathway (10.7 years). However, this is likely due to the method of categorizing girls, in that girls who are classified as asynchronous are more likely to have early development of one marker (breast for girls in the thelarche group, and pubic hair for girls in the pubarche group) compared to the average age at development for the whole cohort. Similarly, synchronous girls are more likely to have later ages at initiation, because on average for the whole cohort, breast development occurs before pubic hair development. However, there is a biological basis for later onset of breast development among girls in the pubarche group, since adrenal androgens may have an inhibitory effect on ovarian estrogen production [29]. 

The median age at menarche in this cohort was 12.87 years, similar to earlier reports [6, 30]. However, age at menarche varied by puberty pathway; girls in the pubarche pathway had an older age at menarche compared to girls in the synchronous and thelarche pathways. In addition, a girl's BMI at 8 years of age, ethnic background, maternal prepregnancy BMI, and birth order were associated with initiation pathway. These findings may relate to differences in the actions of the endocrine and central nervous system, which differentially regulate breast and pubic hair development. That is, some of these factors may act on the hypothalamic pituitary gonadal axis (and subsequent breast development), the hypothalamic pituitary adrenal axis (and subsequent pubic/axillary hair development), or both. Other studies have also reported an association between increased weight and accelerated pubertal development [31] and noted that breast development may be more sensitive to body composition than pubic hair development due to differences in the regulatory pathways for the two processes. Race and ethnicity are also known to be associated with timing of puberty [1, 2, 5, 32, 33]. However, the small number of nonwhite girls and the heterogeneity of this group limit comparisons by race. The relationship of initiation pathway with maternal prepregnancy BMI may be due to the correlation between maternal and child BMI [34, 35], as seen by the attenuation of the effect of maternal BMI in the model including both maternal and child BMI variables. The association between maternal weight, child weight, and pubertal development may be due to a combination of shared genetic and environmental factors. Finally, birth order has been reported to be associated with age at menarche, with first born girls generally having their first period at a younger age compared to later born girls [36, 37]. Although the reasons for this pattern are not known, they may relate to differences in birth intervals, and changing parental care practices. 

There are some limitations in this analysis. Questionnaires were sent on a yearly basis; the long interval between assessments means that critical stages and transitions may not have been captured for each individual. Consequently, some girls may have been misclassified with regards to initiation pathway (i.e., entry into breast or pubic hair stage 2 may have occurred shortly after the completion of a questionnaire). However, with our large sample size and use of interval-censored survival analysis, we were able to estimate ages at transition for the cohort with good precision. We did not adjust for multiple comparisons in our modeling procedure, although the variables remaining in the multivariate model would have been eligible even using more stringent inclusion criteria. Not all of the eligible ALSPAC participants provided information on pubertal development, and most did not provide information every year the questionnaire was administered. To assess the potential for selection bias, we compared selected sociodemographic characteristics of participants who returned at least one Growing and Changing questionnaire, to those who did not. Respondents were more likely to have mothers with higher education (74.5% with an O-level or higher, compared to 59.5%), and mothers with an “upper” social class (39.7% compared to 28.6%). In addition, respondents were less likely to be of nonwhite race/ethnicity (4.0% compared to 5.9%). These differences may have impacted our findings; based on observed differences, respondents may be more likely to experience later maturation compared to the whole cohort, leading to overestimates of age at pubertal milestones and underestimation of association between sociodemographic characteristics and pubertal characteristics. 

The Growing and Changing questionnaire could be completed by the parent, child, or both. For earlier ages, the proportion of questionnaires completed by the parent alone was higher, while for later ages the proportion completed by the child alone was higher. However, a previous study reported good correlation between maternal and child assessments of breast and pubic hair stage [38]. Finally, developmental data were self-reported and may not accurately reflect a girl's stage of breast and pubic hair development. One possibility is that girls categorized as having synchronous initiation of breast and pubic hair development had more subtle physical manifestations, which were not noticed until a more advanced stage. Also, adipose tissue may be mistaken for breast development upon visual inspection. Thus, some overweight or obese girls may have been misclassified in terms of breast development stage, and therefore initiation pathway. Among the girls who regressed in breast stage, a higher proportion was categorized as obese or overweight at 8 years of age, compared to the entire cohort (20% versus 10.4%, and 15.9% versus 12.4%, resp.). This may indicate that some girls reported too high a stage of breast development and reported “regressing” in stage after a weight loss or redistribution of adipose tissue. However, among obese girls included in these analyses, reported breast development progressed over time, which suggests true development of breast tissue as opposed to adipose tissue. Further, when stratifying by BMI group, girls who were overweight or obese had an earlier age at entry into both breast and pubic hair development compared to girls who were underweight or normal weight. Since pubic hair stage is not likely to be affected by adiposity, an advancement in the age at beginning pubic hair development reinforces the possibility that heavier girls are indeed maturing more quickly. 

Strengths of this analysis include the longitudinal design, large sample size, and representative nature of the cohort. Repeated assessments of girls' development allowed the estimation of initiation pathway, and ages at initiation and at menarche.

## 5. Conclusions

Our findings confirm that girls experience different initiation pathways to development of secondary sexual characteristics, and that pathway may be related to various maternal and child characteristics. Our findings also support a general trend towards earlier maturation, which may be due to factors including increasing prevalence of overweight and obesity, and environmental exposures. Further research will be needed to determine causal relationships for observed associations, as well as impact on future health outcomes.

## Figures and Tables

**Table 1:** Median age^*a*^ at Entry into tanner stage ≥2 of breast and pubic hair development and at menarche, overall and by initiation pathway.

	Breast		Pubic Hair		Menarche	
Group (*n*)^b^	Age (95% CI)	Quartiles	Age (95% CI)	Quartiles	Age (95% CI)	Quartiles
All girls (3938)	10.19 (10.14, 10.24)	9.16–11.22	10.95 (10.90, 11.00)	9.99–11.91	12.87 (10.82, 12.91)	12.08–13.65
Synchronous (1631)	10.65 (10.58, 10.72)	9.81–11.49	10.65 (10.58, 10.72)	9.81–11.49	12.84 (12.78, 12.91)	12.06–13.63
Thelarche (1482)	9.43 (9.35, 9.51)	8.42–10.44	11.63 (11.57, 11.70)	10.86–12.40	12.78 (12.71, 12.85)	12.00–13.57
Pubarche (408)	11.31 (11.19, 11.44)	10.52–12.10	9.44 (9.28, 9.59)	8.42–10.46	13.13 (13.00, 13.26)	12.34–13.92

^a^Normal distribution specified.

^b^Girls with unknown initiation pathway (*n* = 417) were randomly allocated to the initiation pathway groups (proportionate to the distribution among girls with known pathway) and included in the survival models for age at entry into breast and pubic hair stage 2.

**Table 2:** Characteristics of study participants, overall and by initiation pathway.

Characteristic^a^	All girls	Thelarche	Pubarche	Synchronous	*P*-value^b^
	N (%)	N (%)	N (%)	N (%)	

Total	3938 (100)	1482 (100)	408 (100)	1631 (100)	
Mother's highest education					.03
CSE/none	583 (15.4)	212 (14.8)	43 (10.7)	243 (15.7)	
Vocational	351 (9.3)	116 (8.1)	29 (7.2)	158 (10.2)	
O-level	1331 (35.2)	515 (35.9)	147 (36.7)	539 (34.8)	
A-level	936 (24.8)	374 (26.1)	103 (25.7)	372 (24.0)	
Degree	579 (15.3)	217 (15.1)	79 (19.7)	236 (15.3)	
Mother's prepregnancy BMI					<.0001
<18.5	186 (5.2)	56 (4.1)	15 (4.0)	94 (6.4)	
18.5–24.9	2671 (75.1)	1007 (74.4)	314 (84.2)	1075 (73.5)	
25.0–29.9	516 (14.5)	208 (15.4)	37 (9.9)	217 (14.8)	
≥30.0	183 (5.2)	83 (6.1)	7 (1.9)	76 (5.2)	
Mother's age at delivery					.02
<20 years	103 (2.6)	37 (2.5)	4 (1.0)	50 (3.1)	
20–24 years	620 (15.7)	240 (16.2)	43 (10.5)	252 (15.5)	
25–29 years	1587 (40.3)	585 (39.5)	187 (45.8)	174 (39.1)	
≥30 years	1628 (41.3)	620 (41.8)	174 (42.7)	692 (42.4)	
Birth order					.01
First born	1326 (34.6)	540 (37.1)	155 (38.8)	515 (32.6)	
Second born	1277 (33.3)	485 (33.4)	137 (34.3)	531 (33.6)	
Third born or later	1235 (32.2)	429 (29.5)	108 (27.0)	535 (33.8)	
Child ethnic background					.0004
White	572 (96.0)	1377 (97.6)	374 (94.4)	1447 (95.0)	
Nonwhite	148 (4.0)	34 (2.4)	22 (5.6)	77 (5.0)	
BMI at age 8					<.0001
<5th percentile	69 (2.5)	20 (1.8)	12 (3.7)	29 (2.6)	
5th–85th percentile	2096 (74.7)	740 (65.8)	285 (86.9)	868 (79.0)	
85th–95th percentile	348 (12.4)	182 (16.2)	23 (7.0)	121 (11.0)	
≥95th percentile	293 (10.4)	183 (16.3)	8 (2.4)	81 (7.4)	

^a^Information was missing for some girls, including information on mother's education (*n* = 158, 4.0%), mother's age at menarche (*n* = 538, 13.7%), mother's social class (*n* = 738, 18.7%), mother's prepregnancy BMI (*n* = 382, 9.7%), sugar in urine at any point during pregnancy (*n* = 362, 9.2%), breastfeeding (*n* = 141, 3.6%), girl's birth order (*n* = 100, 2.5%), girl's birthweight (*n* = 53, 1.4%), girl's race/ethnicity (*n* = 218, 5.5%), girl's height at age 8 (*n* = 1039, 26.4%), and girl's BMI at age 8 (*n* = 1132, 28.8%).

^b^ *P*-value is from univariate logistic regression models.

**Table 3:** Polytomous logistic regression model for initiation pathway.

Characteristic	Thelarche versus Synchronous OR (95% CI)	Pubarche versus Synchronous OR (95% CI)	Pubarche versus Thelarche OR (95% CI)
BMI at age 8			
<5th percentile	0.79 (0.41, 1.49)	1.24 (0.58, 2.64)	1.58 (0.71, 3.55)
5th–85th percentile	Reference	Reference	Reference
85th–95th percentile	**1.82 (1.39**, **2.38)**	0.67 (0.41, 1.07)	**0.37 (0.23**, **0.58)**
≥95th percentile	**2.65 (1.95**, **3.60)**	**0.29 (0.12**, **0.67)**	**0.11 (0.05**, **0.25)**
Child ethnic background			
White	Reference	Reference	Reference
Nonwhite	**0.47 (0.27**, **0.80)**	1.55 (0.87, 2.77)	**3.23 (1.71**, **6.45)**
Mother's prepregnancy BMI			
<18.5	**0.64 (0.42**, **0.99)**	0.68 (0.37, 1.24)	1.05 (0.55, 1.99)
18.5–24.9	Reference	Reference	Reference
25.0–29.9	0.90 (0.68, 1.15)	**0.60 (0.39**, **0.94)**	0.68 (0.44, 1.06)
≥30.0	1.13 (0.74, 1.73)	0.35 (0.13, 1.01)	**0.31 (0.11**, **0.89)**
Birth order			
First born	Reference	Reference	Reference
Second born	0.82 (0.66, 1.01)	0.95 (0.69, 1.29)	1.16 (0.85, 1.58)
Third born or later	**0.72 (0.57**, **0.90)**	0.74 (0.53, 1.03)	1.03 (0.74, 1.44)
